# Reply to Gringras et al. Comment on “Paditz et al. The Pharmacokinetics, Dosage, Preparation Forms, and Efficacy of Orally Administered Melatonin for Non-Organic Sleep Disorders in Autism Spectrum Disorder During Childhood and Adolescence: A Systematic Review. *Children* 2025, *12*, 648”

**DOI:** 10.3390/children12101362

**Published:** 2025-10-09

**Authors:** Ekkehart Paditz, Bertold Renner, Rainer Koch, Barbara M. Schneider, Angelika A. Schlarb

**Affiliations:** 1 Centre for Applied Prevention/Zentrum fur Angewandte Prävention, 01307 Dresden, Germany; 2 Institute of Clinical Pharmacology, Faculty of Medicine Carl Gustav Carus, Dresden University of Technology, 01069 Dresden, Germany; bertold.renner@tu-dresden.de; 3 Formerly Institute for Medical Informatics and Biometry, Faculty of Medicine Carl Gustav Carus, Dresden University of Technology, 01069 Dresden, Germany; rainer.koch.01@gmx.de; 4 Paediatric Sleep Medicine Centre in Foundation, 83549 Landshut, Germany; b.schneider@kinder-schlaf.de; 5 Faculty of Psychology and Sports Science, Bielefeld University, 33615 Bielefeld, Germany; angelika.schlarb@uni-bielefeld.de

We sincerely thank Drs. Gringras, Malow, and Schröder for their comments [1] on The Pharmacokinetics, Dosage, Preparation Forms, and Efficacy of Orally Administered Melatonin for Non-Organic Sleep Disorders in Autism Spectrum Disorder (ASD) During Childhood and Adolescence: A Systematic Review [2]. Their comments raise important questions: Are we applying new knowledge in the literature analyses, thereby becoming a learning system capable of reviewing existing data from different perspectives? Furthermore, are we prepared for a critical discourse that highlights gaps in current research, or are we merely reiterating studies which, from today’s perspective, require redesigned trials to address methodological flaws that limit their impact?

(1) **Consistent Application of Inclusion Criteria**

The selected studies in the systematic review by Paditz et al. (2025) [2] fulfilled the stated inclusion criteria. The inclusion criteria were RCTs testing the efficacy of melatonin treatment in children and adolescents with ASD if (a) ASD was specified as the sole diagnosis or (b) if a subgroup analysis was performed in the presence of psychiatric comorbidities or other diagnoses.

Regarding the statement on ASD outcome measures, avoiding the use of descriptive criteria, e.g., weight or attention deficit hyperactivity disorder (ADHD), limits new perspectives [3] that are essential for better clinical understanding. Therefore, we only partly agree with Gringras et al. While sleep-specific outcome measures (e.g., sleep onset latency or total sleep time) are essential, clinical core characteristics of the study population with an ASD diagnosis in the context of pharmacokinetics such as sex, age, weight, and additional factors like height, intellectual disability, or co-occurring ADHD, are equally important, even though “they are not ASD-specific outcomes”. These variables should be considered core clinical descriptors of subtypes within the autism spectrum. For example, height allows BMI calculations, intellectual disability allows the degree of potential trial adherence to be reviewed; and ADHD diagnosis allows restlessness to be reviewed, which may indicate an organic cause of insomnia and require a different treatment [4].

(2) **Inclusion of Key Studies and Precise Interpretation of Existing Evidence**
  The excluded RCTs from Gringras et al. (2012) [5], Appleton et al. (2012) [6], and Gringras et al. (2017) [7] were excluded because the subgroup analysis was not sufficient:
  (a) The study from 2012 included a high level of heterogeneity (“neurodevelopmental diseases”; N = 146) without further subgroup analysis. Aside from the already referenced and analyzed 60 patients with ASD whose comorbidities were not listed, the group of 146 patients contained subgroups of 22 patients with the main diagnoses of developmental delays, 13 patients with epilepsy, and 3 additional patients with both epilepsy and ASD, as well as 48 patients with unspecified diagnoses, which makes a clean subgroup analysis impossible. It may have been possible if a table was provided with all co-morbidities and a biostatistical analysis utilizing this information. The provided data in Figure 1 [Baseline characteristics of children with neurodevelopmental disorders in the study of effect of melatonin on sleep problems] does not include core mental health comorbidities for children with ASD, such as anxiety or ADHD, both affecting sleep. We see this missing information as a significant methodological flaw.
  (b) The study of Appleton et al. (2012) [6] described and published the results for the same data set (Table 4 in Appleton 2012 = Table 1 in Gringras et al. 2012) [5,6]. In this article, some conditions are described in more detail: “The population studied was a heterogeneous group comprising a large number of children with a wide range of neurological and developmental disorders, including those with specific genetic disorders but also those without a specific genetic or syndromic diagnosis.” [6]; “five patients had Down syndrome, one had DiGeorge syndrome, one had Cornelia de Lange syndrome and one had Smith–Magenis syndrome” (p. 34) [6]. This confirms our statement above. However, these studies were previously analyzed and in the current paper they have also been referenced in Suppl. 1 [2], so the conclusion that they were “excluded without explanation” is incorrect.
  (c) The heterogeneity of the quality of studies available to date, including the ‘blind spots’ caused by the lack of or insufficient consideration of several quantitative and qualitative characteristics, was intensively discussed and documented at the 2024 annual meeting of the International Paediatric Sleep Association (IPSA) in Glasgow (Figure 1) [8,9]. Jorgensen et al. have come to the same conclusion regarding the use of melatonin in children and adolescents with neurogenetic disorders: “… previous reviews had important limitations. The conclusions were affected by a high degree of heterogeneity, high and unclear risk of bias in the included trials and small sample sizes.” [10]. McWilliams et al. have similarly pointed out in relation to studies on ADHD that characteristics that characterize sleep have not yet been taken into account in sufficient quantity and quality: “Polysomnography and actigraphy used a heterogeneous spectrum of sleep-related variables and technical algorithms, respectively. 19/23 sleep questionnaires [only] were validated covering a spectrum of sleep-related domains. Despite the intrinsic nature of sleep disturbances in ADHD, the number of RCTs measuring sleep-specific outcomes is limited and tools to measure outcomes are not standardized. Given the potential adverse effects of ADHD medications on sleep, sleep should be included as a core outcome measure in future clinical trials.” [11].
  (d) Regarding potential industry bias, the statement of Dr. Gringras that the authors of Paditz et al. (2025) [2] have a confounded view, on the grounds that the Gringras’s studies were more “scrutinized” than others, cannot be upheld for the following reasons:
    - The study by Hayashi et al. (2022) was referenced with the quotation “The present study was funded by Nobelpharma Co., Ltd.” [2,12].
    - The study by Gringras et al. (2017) was not only funded “…by Neurim Pharmaceuticals” but also the data were analyzed by Neurim (“Mr. Breddy served as the statistical expert for this research and was paid by Neurim Pharmaceuticals.”) [7]. Furthermore, the detailed list of disclosures reveals that two of the authors had a position as a “chief investigator and consultant for Neurim Pharmaceuticals” and two were employees of Neurim Pharmaceuticals. Given the methodological limitations stated above, a large cohort study involving multiple industry-funded researchers with conflicts of interest usually raises academic concerns.
(3) **Available Pharmacokinetic Data for Children**
(a) Gringras et al. (2012) [5] reference an EMA report, which Paditz et al. (2025) [2] have also referenced. To clarify, the EMA report does not contain any pharmacokinetics data on children and adolescents with ASD but refers to Circadin (R), a prolonged-release formulation of melatonin used to treat insomnia in adults, particularly those with depression, aged 55 and older.
(b) Schröder et al. (2021) [13] did not provide any further data on pharmacokinetics (see also EMA report (p. 44): “Children and Race. No studies have been provided”).

The statement “omission…” is not only strong but also factually incorrect.

(4) **Claims about Dose–Response Data**

As stated, the EMA report does not contain any pharmacokinetic data on children and adolescents, and, in particular, on children and adolescents with ASD (see point 3 above). Additionally, Schröder et al. (2021) [13] did not provide any further pharmacokinetic data. In that context, the conclusion by Paditz et al. (2025) was as follows: “To our knowledge, dose–response correlations of melatonin and sleep in children and adolescents with ASD [=ASS] have only been investigated by Goldman et al. in an observational study involving nine children [14].” [2]. The statement made by Gringras et al. that “The claim that dose–response relationships have only been investigated in observational cohorts involving nine children is incorrect,” is also not only strong, but also explicitly incorrect.

(5) **Clinical Relevance and Generalisability**

The listed clinically meaningful outcomes are important and have been referenced multiple times, e.g., Table 1, Figure 5 [2]. Therefore, this statement is also not only strong, but explicitly incorrect. The statement that the consensus statement by Kotagal et al. (2024) [15] was not integrated in the discussion is correct and we accept the critique, although the subject of our systematic review was not the listing of guidelines but the collection and analysis of RCTs. It remains unclear why Kotagal et al. only included studies from 2012 onwards, even though clinical applications of melatonin in children had already been presented by McArthur in 1998 [16] and Palm et al. in 1991 and 1997 [17,18]. The group led by Bruni et al. shares our view: “To date, there are no specific guidelines for the treatment of insomnia in children with ASD and/or ADHD.” [19].

(6) **Statements Regarding Regulatory Guidance and Recommendations**

Currently, there are no multi-arm RCTs evaluating the efficacy of rapid-release and prolonged-release melatonin preparations against placebo in children and adolescents. As stated multiple times, a knowledge gap exists regarding non-organic pediatric sleep disorders in individuals with ASD. The need for further research has been clearly addressed, and we agree with Gringras and Schröder that the dosage of melatonin for non-organic sleep disorders in children and adolescents should be age- and diagnosis-specific (Paditz et al. (2025), Chapter 4.1: ‘Importance of Diagnosis-Related RCTs with Subgroup Analyses’) [2].

The status of regulatory approval for melatonin preparations was not the subject of our systematic review of RCTs in which melatonin was analyzed in children and adolescents with non-organic sleep disorders in ASD. We will respond briefly to the claim made by Gringras, Malow, and Schröder that we summarized different statements and made accusations. It is correct that the prolonged-release preparation Slenyto^®^ has been approved by the EMA, in Switzerland and in Australia. In Germany, however, a legally regulated multi-stage procedure has been established in which, among other things, criteria of evidence-based medicine and health economic analyses are taken into account at the level of the Joint Federal Committee (G-BA) and the Institute for Quality and Efficiency in Health Care (IQWiG) [20,21]. In 2019 and 2024, the IQWiG criticized the preparation Slenyto^®^, among other things, because the studies submitted by Gringras et al. in 2017 [7] did not continue sleep hygiene measures and behavioural therapy interventions DURING treatment with melatonin. The G-BA revoked its 2019 benefit assessment for Slenyto^®^ in 2024. The ‘Arzneiverordnungsreport 2024’ (Drug Prescription Report 2024) by Ludwig et al. 2025, which is particularly relevant for health policy decision-makers in Germany, explicitly refers to this IQWiG report [22]. This decision has practical consequences for the doctors and pharmacists involved, as they are obliged to prescribe and dispense the most cost-effective melatonin preparation, with the exception of justified individual cases [20]. The view that psychagogic measures should also be continued during treatment with melatonin is not shared by Gringras, as shown in a recent interview: “So, should melatonin be used to help children sleep? When used as a prescription medicine after behavioural interventions have not resolved sleep problems, Gringras calls melatonin “the safest and the best sleep treatment to date.” [23]. We emphasize, consistent with the IQWiG assessments cited above, that behavioural measures should continue and be optimized during any pharmacological treatment rather than being discontinued once melatonin is started. Continued non-pharmacological care maximizes benefit and allows for better evaluation of treatment effect.

(7) **Terminology and Clinical Insight**

We appreciate this comment but assume that our readers understand the difference between “delayed” and “non-delayed” in this context correctly, even if there are specific technical terms for modified-release products. For drugs with prolonged release of the active ingredient, the terms ‘delayed release’ or ‘sustained release’ are used (Ronchi et al. (2019); Hu et al. (2025)) [24,25]. As of 2 August 2025, 1732 publications on ‘delayed release’ can be found in PubMed [Title/Abstract]. More importantly, Gringras does not use the terminology he requires consistently: “There is a wide variety of unlicensed fast-release, slow release, and liquid preparations of melatonine…A prolonged-release formulation of melatonin (Circadin) was licenced in 2008 as a short-term treatment…” (Gringras 2025, p. 171) [26].

**Figure 1:**
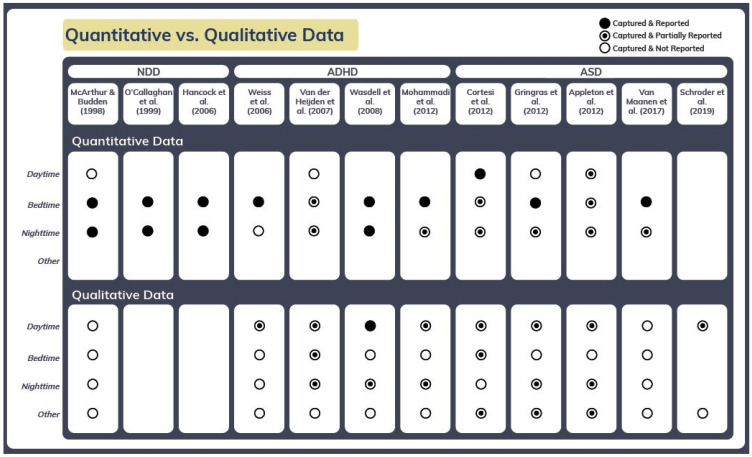
Heterogeneity of the quality of studies available to date. Heterogeneity of the quality of existing randomized studies on neurogenetic disorders, ADHD, and ASD [8]. The limitations of the studies by Gringras et al. 2012 [5] and Appleton et al. 2012 [6] have been explicitly pointed out. Van Maanen, et al. (2017) [27], Schroder, et al. (2019) [28] and [16,29,30,31,32,33,34,35].

## Conclusions

Drs. Gringras, Mallow, and Schröder submitted their comments in the interest of maintaining scientific rigour and transparency in reviews that inform clinical practice. As initially stated, we are thankful that they are initiating this necessary discussion on the quality of reviews, which we would like to extend to the design of RCTs. While the RCTs by Gringras et al. (2012, 2017) [5,7] had some methodological limitations that, within a learning system, could have helped to improve further RCT design, later study designs did not show a clear advancement in methodologies. Finally, while we highly value critical academic discourse, we recommend using less misplaced language and avoiding incorrect claims.
